# Application of pattern analysis in fine needle aspiration of solitary nodule of thyroid

**DOI:** 10.4103/0970-9371.66688

**Published:** 2010-01

**Authors:** Jyothi B Lngegowda, Prakash H Muddegowda, Kumar N Rajesh, Kurpad R Ramkumar

**Affiliations:** Department of Pathology, VMKV Medical College, Salem, India

**Keywords:** Fine needle aspiration, pattern analysis, solitary thyroid nodule

## Abstract

**Background:**

Various methods are used to arrive at a conclusive diagnosis of thyroid lesions on fine needle aspiration cytology (FNAC). Systemic pattern analysis is one such that can be used to analyze the lesions and divide them into individual categories.

**Aims:**

To study the application of pattern analysis in the interpretation of solitary thyroid nodule (STN).

**Materials and Methods:**

Two hundred and nineteen cases of fine needle aspiration cytology of STN were reviewed along with histopathological correlation. Smears were classified based on primary and secondary patterns. Predominant pattern (primary) was identified and lesion categorized. This was followed by identifying the next dominant pattern (secondary) and recategorization. Cytological diagnosis based on primary and secondary patterns was correlated with the histopathological diagnosis.

**Results:**

Based on pattern analysis, the study had a sensitivity of 66.7% and specificity of 98.9%. The positive predictive value and negative predictive value were 88.9% and 96% respectively and the overall diagnostic accuracy was 95.4%.

**Conclusions:**

The present study demonstrates the feasibility and applicability of pattern analysis in diagnosing thyroid lesions by FNAC, which could be easily reproducible.

## Introduction

Fine needle aspiration cytology (FNAC) is now accepted as a cost-effective procedure in the initial assessment and management of thyroid enlargement. It is minimally invasive, has a low complication rate and offers non-operative diagnosis for most of the thyroid lesions. It has been shown that FNAC is highly successful in triaging patients with solitary thyroid nodule (STN) into operative and non-operative groups.[1,2]

Clinically detectable thyroid nodules occur in 4–10% of the population. The majority of the nodules are non-neoplastic. Among the neoplastic lesions, only 5–30% are malignant and require surgical intervention. The main goal of thyroid FNA is to identify the STN that require surgery and reduce the overall number of thyroidectomies for patients with benign disease.[3]

Several studies have confirmed the accuracy and reliability of FNAC in the differentiation between relatively small number of malignant thyroid nodules and the larger percentage of nodules that are benign.[4]

The numerous diagnostic procedures currently available improve the anatomic, pathologic, radiological and functional assessment of thyroid nodules but may also lead to unjustified increase in cost with little practical gain, if not used rationally. As most of the hospitals lack some of these ancillary diagnostic investigations, FNAC is still regarded as the single most accurate and cost-effective procedure.[5]

Despite the many sites and many types of tumours that are aspirated, there are a limited number of patterns that are observed in the aspirated material. However, the frequency, significance and difference of each pattern varies with the site.[6]

Many authors have used various methods to arrive at a conclusive diagnosis of thyroid lesions on FNA. Here, we propose a partially modified method based on pattern analysis, as suggested by Andrew A. Renshaw,[6] to analyse the lesions and divide them into individual categories.

## Materials and Methods

Material for the present study was obtained retrospectively over a period of four years from the archives of the Department of Pathology, VMKV Medical College, Salem. Majority of the aspirations were performed in the department itself by cytopathologists. Before aspiration, physical examination of the thyroid gland was carried out to assess its size, mobility during deglutition, its nodularity and evidence of clinical signs of thyrotoxicosis. Neck nodes were also palpated for enlargement.

Aspiration was performed using disposable 10 ml syringe with a 23 G needle and syringe holder. Non-aspiration technique was also used in some cases to reduce contamination of specimen. The number of passes was kept to a minimum to minimize hemorrhage, and was usually two to three. Each case had a minimum of three smears and was stained with hematoxylin and eosin (H and E), papanicolaou (PAP) and giemsa stains.

A total of 1,346 thyroid aspirations were performed during this period, of which 359 cases were of clinically detected STN, with 233 cases having a follow-up surgery with histopathological correlation. These latter cases were taken for this study. Cases in which sample was inadequate were excluded from the study. In total, 219 cases formed the crux of the study.

Each slide was examined by two different cytologists with a difference in experience of 15 years. They were asked to identify the patterns [Table 1] and make a provisional diagnosis.

**Table 1:** List of various patterns used in this study

Group	Primary pattern	Secondary pattern	Observation
I	Colloid-rich background	Hurthle cell rich	Predominant thick or thin colloid with Hurthle cell >follicular cells
		Macrophage rich	Predominant thick or thin colloid with increased macrophages
		Microfollicle poor	Predominant thick or thin colloid with sheets/scattered follicular cells
		Microfollicle rich	Predominant thick or thin colloid with plenty of microfollicles
II	Biphasic	Lymphocytic background	Combination of follicular cells, Hurthle cells with polymorphous population of lymphocytes
		Hurthle cell rich	Combination of follicular cells, Hurthle cells with Hurthle cells >follicular cells/other cells
III	Hurthle cell rich	Colloid-rich background	Hurthle cells >follicular cells/other cells in predominant colloid background
		Hemorrhagic background	Hurthle cells >follicular cells/other cells in an hemorrhagic background
		Lymphocytic background	Hurthle cells >follicular cells/other cells with polymorphous population of lymphocytes
IV	Macrophage rich	Colloid-rich background	Macrophage >follicular cells/other cells with predominant colloid background
		Hurthle cell rich	Macrophage >follicular cells/other cells with Hurthle cells >follicular cells
		Monotonous crowding	Macrophage >follicular cells/other cells with crowded, overlapping follicular cells
		Microfollicle poor	Macrophage >follicular cells/other cells with sheets/scattered follicular cells
		Microfollicle rich	Macrophage >follicular cells/other cells with plenty of microfollicles
V	Monotonous crowding	Enlarged oval nucleus	Crowded, overlapping follicular cells with large cell, bigger than lymphocyte/follicular cell with pale oval nucleus and characteristic nuclear features
VI	Microfollicle poor	Colloid-rich background	Sheets/scattered follicular cells in a predominant colloid background
		Hurthle cell rich	Sheets/scattered follicular cells with Hurthle cells >follicular cells/other cells
		Macrophage rich	Sheets/scattered follicular cells with Macrophage >follicular cells/other cells
VII	Microfollicle rich	Colloid-rich background	Plenty of microfollicles in a predominant colloid-rich background
		Hemorrhagic background	Plenty of microfollicles in a highly hemorrhagic background
		Macrophage rich	Plenty of microfollicles with macrophage >follicular cells/other cells
		Enlarged oval nucleus	Plenty of microfollicles with large cell, bigger than lymphocyte/follicular cell with pale oval nucleus and characteristic nuclear features
VIII	Pleomorphic	Hemorrhagic background	High-grade cytological change in a highly hemorrhagic background
		Amyloid background	High-grade cytological change with background composed of amyloid/pink fibrillary material

The final provisional diagnosis was given out in the following manner:[1]

1. Benign
2. Atypia of undetermined significance (AUS)
3. Neoplasm
4. Suspicious for malignancy
5. Malignant

The cytologists were asked to identify the predominant pattern (primary) first and then give out a diagnosis and then to identify the next dominant pattern (secondary) and give the combined pattern diagnosis. The variation between primary, secondary pattern and final cytological diagnosis was matched with the final histopathological diagnosis for correlation and statistical data were prepared.

### Statistical Analysis

For each correlation, sensitivity, specificity, positive predictive value, negative predictive value and diagnostic accuracy were calculated. Interobserver variation was assessed by kappa measure of agreement.

## Results

Two hundred and nineteen (219) cases of STN aspirates with histopathological confirmation were evaluated by two observers based on the primary and secondary patterns. For convenience of discussion, the lesions were categorized into eight (8) groups based on primary pattern, as shown in Table 2.

**Table 2:** List of cases with cytohisto correlation with application of pattern

Group	No. of cases	Primary pattern	Secondary pattern	Provisional diagnosis	Histopathological diagnosis
I	3	Colloid-rich background	Hurthle cell rich	Benign	Colloid goitre (3)
	7	Colloid-rich background	Macrophage rich	Benign	Colloid goitre (7)
	28	Colloid-rich background	Microfollicle poor	Benign	Colloid goitre (28)
	5	Colloid-rich background	Microfollicle rich	Atypia of undetermined significance	Adenomatoid nodule (2) Colloid goiter (1) Follicular adenoma (2)
II	13	Biphasic	Lymphocytic background	Benign	Hashimotos thyroiditis (13)
	4	Biphasic	Hurthle cell rich	Neoplasm	Hurthle cell adenoma (3) Hashimotos thyroiditis (1)
III	2	Hurthle cell rich	Colloid-rich background	Benign	Colloid goitre (2)
	5	Hurthle cell rich	Hemorrhagic background	Neoplasm	Hurthle cell adenoma (5)
	6	Hurthle cell rich	Lymphocytic background	Benign	Hashimotos thyroiditis (6)
IV	16	Macrophage rich	Colloid-rich background	Benign	Colloid goitre (16)
	1	Macrophage rich	Hurthle cell rich	Neoplasm	Hurthle cell adenoma (1)
	1	Macrophage rich	Monotonous crowding	Malignant	Papillary carcinoma (1)
	9	Macrophage rich	Microfollicle poor	Benign	Colloid goitre (9)
	4	Macrophage rich	Microfollicle rich	Neoplasm	Follicular adenoma (3) Adenomatoid nodule (1)
V	7	Monotonous crowding	Enlarged oval nucleus	Malignant	Papillary carcinoma (5) Follicular adenoma (2)
VI	33	Microfollicle poor	Colloid-rich background	Benign	Colloid goitre (30) Follicular adenoma (1) Papillary carcinoma (2)
	1	Microfollicle poor	Hurthle cell rich	Neoplasm	Colloid goitre (1)
	13	Microfollicle poor	Macrophage rich	Benign	Colloid goitre (9) Papillary carcinoma (4)
VII	19	Microfollicle rich	Colloid-rich background	Atypia of undetermined significance	Adenomatoid nodule (8) Colloid goitre (7) Follicular adenoma (4)
	29	Microfollicle rich	Hemorrhagic background	Neoplasm	Adenomatoid nodule (1) Colloid goitre (1) Follicular adenoma (25) Papillary carcinoma (2)
	3	Microfollicle rich	Macrophage rich	Atypia of undetermined significance	Colloid goitre (2) Adenomatoid nodule (1)
	4	Microfollicle rich	Enlarged oval nucleus	Malignant	Papillary carcinoma (4)
VIII	4	Pleomorphic	Hemorrhagic background	Malignant	Anaplastic carcinoma (4)
	2	Pleomorphic	Amyloid background	Malignant	Medullary carcinoma (2)

Of the 219 cases, 195 were benign, consisting of nodular colloid goitre (*n*=116), adenomatoid nodule (*n*=13), Hashimotos thyroiditis (*n*=20), Hurthle cell adenoma (*n*=9) and follicular adenoma (*n*=37).

There were 24 malignant lesions, including papillary carcinoma (*n*=18), medullary carcinoma (*n*=2) and anaplastic carcinoma (*n*=4).

In the present study, the total numbers of cases of true positives (malignant) were 16, true negatives (benign) were 193, false positives were two and false negatives were eight.

Based on pattern analysis, the statistics for benign and malignant lesions were sensitivity of 66.7%, specificity of 98.9%, positive predictive value of 88.9%, negative predictive value of 96% and diagnostic accuracy of 95.4%.

### Interobserver variation

Unanimous agreement between the two reviewers was observed in 187 (85.38%) cases, including 124 benign, 11 AUS, 37 neoplasm and 15 malignancies. Six cases were diagnosed as benign by one reviewer and as suspicious for malignancy or AUS by the other, and all turned out to be malignant lesions histologically. Sixteen cases diagnosed as AUS by reviewer one, were diagnosed as benign or neoplasm by the other, including 12 histologically proven benign lesions and four malignant lesions. Seven cases were diagnosed as neoplasm by one reviewer and as benign by the other, where three cases turned out to be benign, two cases of adenoma and two malignancies on histopathology. Three cases were diagnosed as malignant by one reviewer and as suspicious for malignancy by the other, which turned out to be one case of malignancy and two cases of adenoma on histopathology.

Benign, AUS and neoplasm were categorized under benign lesions, while suspicious for malignancy and malignant were included under malignant lesions. Considering this, the kappa measure of agreement between benign and malignant lesions between the two reviewers was found to be κ = 0.85.

## Discussion

Our findings illustrate the value of applying a systematic pattern analysis to evaluate the thyroid cytology smear. The present study confirms that assessment of pattern, when applying both primary and secondary pattern, has enough accuracy for the surgeon to triage into operative and non-operative cases.

**Figure 1:**
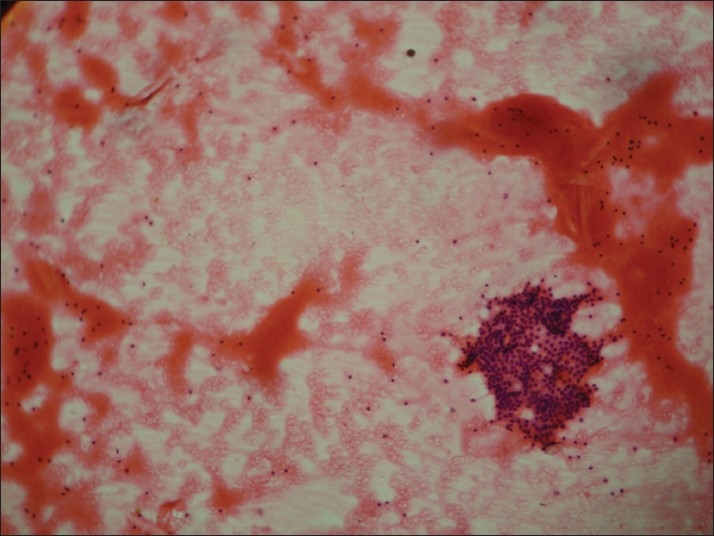
Group I: Colloid-rich background with microfollicle-poor pattern (H and E, ×100)

In Group I [Figure 1], the predominant pattern was colloid-rich background followed by various other secondary patterns, like Hurthle cells (common in colloid goitre), macrophages (cystic lesions) and follicular cells in sheets/scattered/microfollicles. Microfollicles are rarely seen in colloid goitre and need to be differentiated from adenomatoid nodule and follicular neoplasm. In difficult cases, diagnosis of AUS is usually preferred. Macrofollicular adenomas will be usually diagnosed as colloid nodule as more colloid is seen, while thyroid nodules with microfollicles are named as “follicular neoplasm” without knowing the status of encapsulation. Histopathologically, adenomatoid nodule is often incompletely encapsulated, composed of dilated follicles lined with tall cylindrical cells with small follicles and scant or absent colloid. Because of the overlapping features of microfollicles and scant colloid, it is difficult to separate follicular neoplasm from adenomatoid nodule on aspirate.[1,7–9]

**Figure 2:**
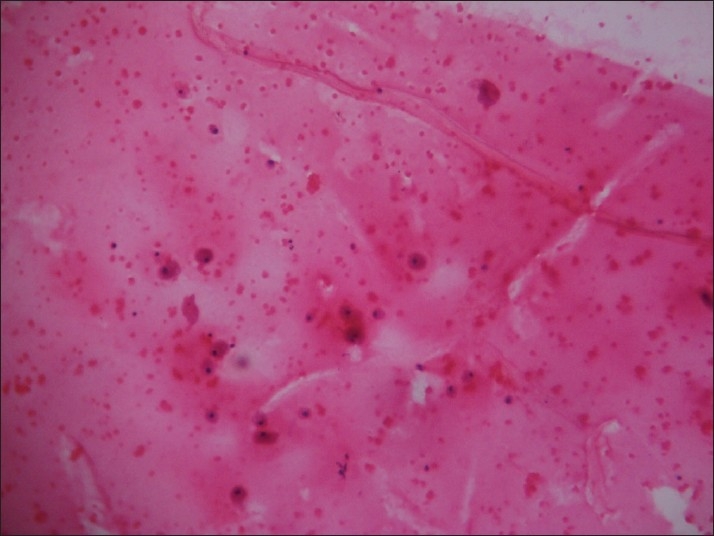
Group II: Colloid-rich background with macrophage-rich pattern (H and E, ×100)

In Group II [Figure 2], the predominant pattern was biphasic, with lymphocyte-rich or Hurthle cell-rich background as secondary patterns. When the Hurthle cells form the major component, diagnosis of neoplasm is usually favored. One case of Hashimotos was grouped in this category because the lesion was a Hurthle cell-rich Hashimotos thyroiditis. Lymphocyte-rich background would have been supportive. Other authors have had a similar experience in this regard.[10]

**Figure 3:**
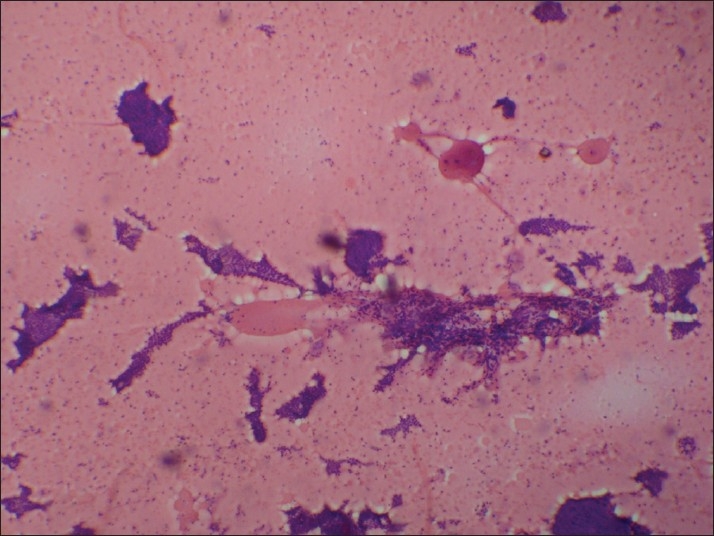
Group III: Microfollicle-poor pattern in a colloid-rich background (H and E, ×100)

In Group III [Figure 3], the predominant pattern was Hurthle cell rich followed by other background patterns, like colloid-rich background (benign diagnosis), hemorrhagic background (neoplasm) and lymphocytic background (lymphocytic/Hashimotos thyroiditis). A hemorrhagic background always points to a highly vascular proliferative lesion and has been shown in other cases in the past, which helped in diagnosing the lesion. If aspirates contain more than 75% Hurthle cells, the possibility of a Hurthle cell neoplasm should be considered, in the absence of which non-neoplastic lesions are to be suspected.[10,11]

**Figure 4:**
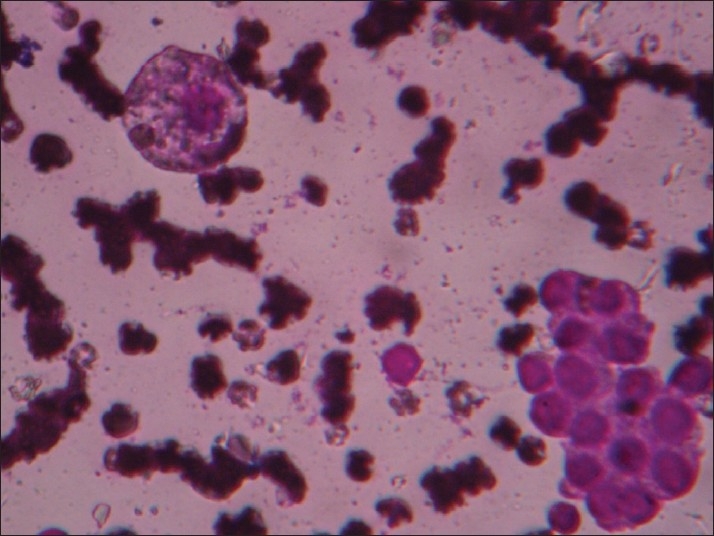
Group IV: Microfollicle-poor pattern with macrophage (H and E, ×400)

In Group IV [Figure 4], the predominant pattern was a macrophage-rich pattern with various other secondary patterns. One case of adenomatoid nodule was inadvertently called as neoplasm; this was probably due to the proliferative nature of the lesion. Cytological differentiation is very difficult in these cases due to overlapping cytological features.[7,9,10,12]

**Figure 5:**
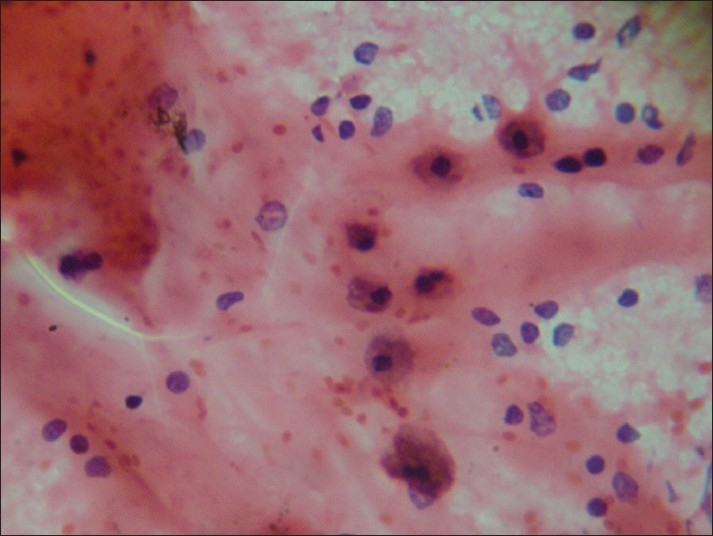
Group V: Macrophage-rich pattern with microfollicle-poor pattern (H and E, ×100)

In Group V [Figure 5], monotonous crowding was the predominant pattern followed by increased cell size and nuclear enlargement (enlarged oval nucleus pattern). Nuclear grooves and intranuclear inclusions were also commonly seen. All cases were reported accurately probably due to the crowding pattern and nuclear details.[1,7] Two cases of follicular neoplasm were overdiagnosed as malignant probably due to increased cellularity and nuclear atypia on cytology.

**Figure 6:**
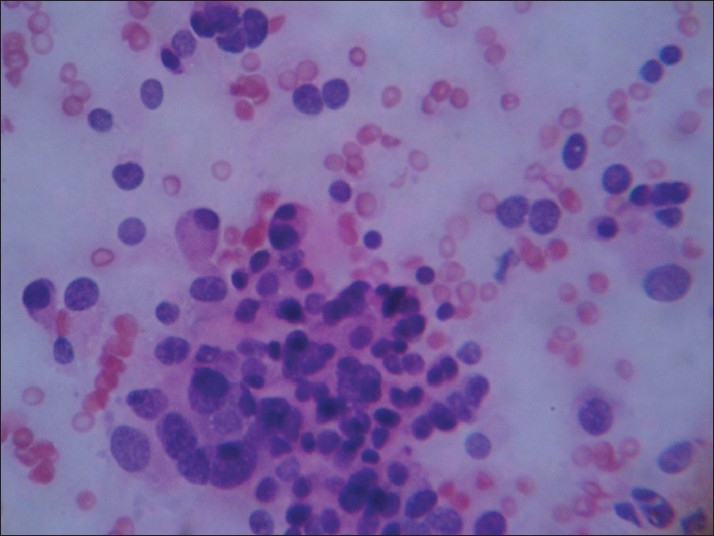
Group VI: Biphasic pattern with lymphocytic background (H and E, ×400)

In Group VI [Figure 6], a microfollicle-poor pattern was predominant, with various secondary patterns. Two cases of papillary carcinoma were missed in a colloid-rich background. Histopathology of these cases showed the presence of microscopic foci of papillary carcinoma in one case and possible cystic change in the other. The cause of detection/diagnosis failure was possibly the presence of small minute foci of a papillary thyroid carcinoma that was missed during aspiration and presence of degenerative foci/interpretation error in another. One case turned out to be follicular adenoma probably due to aspiration performed over the colloid-rich macrofollicular areas of the neoplasm. Similar cases have been encountered by others. Detailed clinical examination and multiple aspirations from different sites would be a possible remedial measure.[1,2,12,13]

**Figure 7:**
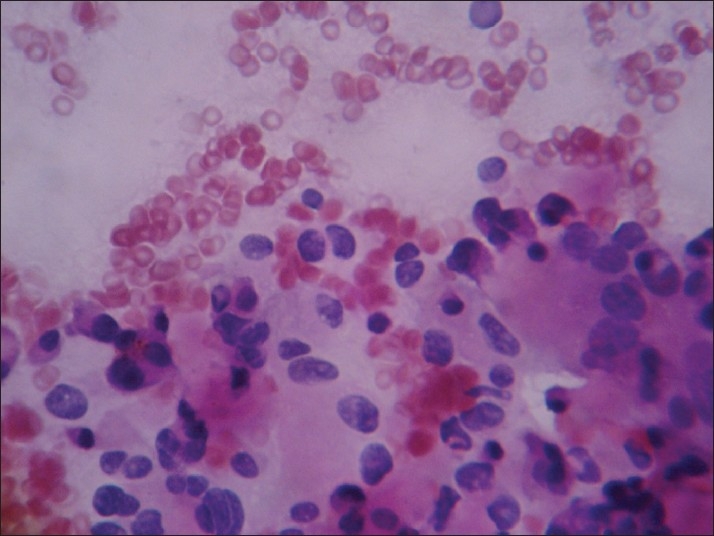
Group VII: Microfollicle-rich pattern in a hemorrhagic background (H and E, ×400)

In Group VII [Figure 7], a microfollicle-rich pattern was predominant. Two cases with hemorrhagic background were diagnosed as neoplasm on FNAC, which turned out to be a follicular variant of papillary carcinoma on histopathology. The presence of follicular structure led to misinterpretation, as has been encountered by others. Four cases with nuclear enlargement (enlarged oval nucleus pattern) were correctly identified as papillary carcinoma. Colloid goitre often forms a part of this group possibly due to difficulty in differentiating between follicular neoplasm and nodular goitre. The most important clue in diagnosing follicular neoplasm is “abundant blood containing rare microfollicles”. Abundant blood is indicative of high microvessel density in the nodule. An exceedingly bloody aspirate containing microfollicles, even if few in number, is a cytologic clue to follicular neoplasm.[12–14]

Performing repeated FNA of persistent nodules is considered by some investigators as a useful way for correcting an initial false-negative diagnosis. Overlapping criteria between adenomatoid nodule and follicular neoplasm could have led to increased diagnosis of AUS on cytology.[3,7,9]

Follicular neoplasm forms a gray zone, with the differential diagnoses including follicular carcinoma, follicular variant of papillary carcinoma (FVPC), follicular adenoma and adenomatoid nodule. Over the past several decades, iodide supplementation to food supplies in many parts of the world has been followed by a corresponding decrease in the incidence of follicular thyroid carcinoma.[5,12]

**Figure 8:**
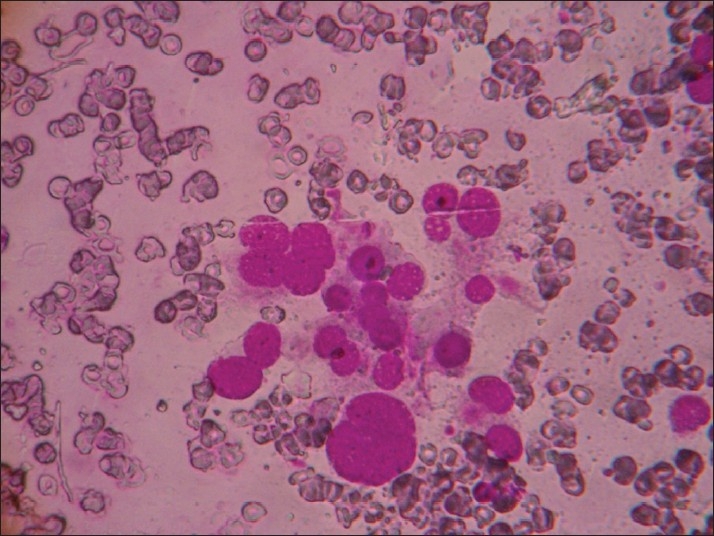
Group VIII: Pleomorphic pattern in a hemorrhagic background (Giemsa, ×400)

In Group VIII [Figure 8], a pleomorphic pattern was predominant, with anaplastic carcinoma being the most common diagnosis. Presence of pink amyloid-like material in the background led to diagnosis of medullary carcinoma in two cases. Presumptive diagnosis of medullary carcinoma of the thyroid based on pleomorphism and amyloid has been described previously. Amyloid must be distinguished from other pink amorphous-appearing material, including fibrin, collagen fibres and non-amyloid kappa light chain protein.[7,15]

AUS is defined as a heterogeneous category that may be appropriate to use for architectural or cytological atypia on a compromised specimen. The risk of malignancy is limited to 5–10%. In the present study, AUS category was high (12.32%), probably due to overlapping findings that were not convincingly benign, but neither sufficient enough to call it as neoplasm/suspicious for malignancy. Limiting the use of this category as suggested by National Cancer Institute (USA) to <7% is desirable because all cases of AUS turned out to be benign on histopathology in our study, thereby reducing the purpose of FNA, which is to limit the number of benign lesions under the scalpel. Studies have shown that eliminating the AUS category increases the false-negative rate.[8,16] The false-negative error rate in our study was 0.93%.

The increased number of AUS in our study might be due to selection of cases having cytohistopathological correlation and increased number of cases being taken for surgery with diagnosis of AUS. We have now suggested follow-up in such cases to our clinicians and to defer surgery till repeat FNAC is performed, as there is an increased likelihood that subtle evidence of malignancy may become obvious during the second FNA and exuberant reactive changes on the initial smear may diminish after several months following first FNA.[16]

The major diagnostic problems are due to use of marginally adequate specimen with diagnosis of malignancy based on just one or two atypical cytological features or overlapping cytological features of various lesions. The efficiency of FNA is responsible for a marked increase in the rate of malignancies found at surgery. The incidence of false-positive error rate for malignancy was zero in the present study. The different statistical values indicating the diagnostic efficacies observed in the present study can be well compared with other studies [Table 3].[3,12,17–21]

**Table 3:** Comparison of results of the present study with other studies performed by standard method

Sensitivity	Present study (pattern analysis) Jyothi *et al*.	Bukhari *et al*.[3]	Lopez *et al*.[19]	Flanagan *et al*.[20]	Chao *et al*.[21]
Specificity	66.7	85	90	81.7	86.1
Specificity	98.9	90	99	56.4	59
Efficacy	95.4	87	99	82	64.6
Clinical reliability	Reliable	Reliable	Reliable	Reliable	Reliable

Considering the data of accuracy, it is evident that pattern analysis is highly accurate and has a low rate of false-negative and false-positive diagnosis.

Our study, to the best of our knowledge, is the first comprehensive systematic attempt to use pattern analysis in the interpretation of FNA cytology in STN.

## Conclusion

FNA cytology is a cost-effective and useful first-line screening and diagnostic technique for the evaluation of STN. Diagnostic accuracy is very high, with a high specificity and sensitivity, thus reducing the number of surgeries. Application of pattern analysis has also allowed the same accuracy and is easily reproducible. We recommend this method in the interpretation of thyroid FNA. Pattern analysis can be applied for diffuse thyroid lesions as well and lesions of other organs too. Our study demonstrates the feasibility and applicability of pattern analysis in interpreting STN lesions.
